# Exploring the vagueness of Religion & Spirituality in complex pediatric decision-making: a qualitative study

**DOI:** 10.1186/s12904-018-0360-y

**Published:** 2018-09-12

**Authors:** Alexandra K. Superdock, Raymond C. Barfield, Debra H. Brandon, Sharron L. Docherty

**Affiliations:** 1 School of Medicine, Duke University, Durham, NC USA; 2 Division of Pediatric Hematology and Oncology, Duke University School of Medicine, 2 Chapel Drive, 0034 Westbrook, Durham, NC 27708 USA; 3 Department of Pediatrics, Duke University School of Nursing, 307 Trent Drive, Durham, NC 27710 USA; 4 School of Nursing, Duke University, 307 Trent Drive, Durham, NC 27710 USA; 5 Pediatrics Residency Program, University of Pittsburgh Medical Center, 4401 Penn Avenue, Pittsburgh, 15224 PA USA

**Keywords:** Pediatric palliative care, Critical care, Neonatology, Life-sustaining therapy, Withdrawal and withholding of treatment, Communication, Religion and medicine, Miracles, Prayer, High-risk newborns

## Abstract

**Background:**

Medical advances have led to new challenges in decision-making for parents of seriously ill children. Many parents say religion and spirituality (R&S) influence their decisions, but the mechanism and outcomes of this influence are unknown. Health care providers (HCPs) often feel unprepared to discuss R&S with parents or address conflicts between R&S beliefs and clinical recommendations. Our study sought to illuminate the influence of R&S on parental decision-making and explore how HCPs interact with parents for whom R&S are important.

**Methods:**

A longitudinal, qualitative, descriptive design was used to (1) identify R&S factors affecting parental decision-making, (2) observe changes in R&S themes over time, and (3) learn about HCP perspectives on parental R&S. The study sample included 16 cases featuring children with complex life-threatening conditions. The length of study for each case varied, ranging in duration from 8 to 531 days (median = 380, mean = 324, SD = 174). Data from each case included medical records and sets of interviews conducted at least monthly with mothers (*n* = 16), fathers (*n* = 12), and HCPs (*n* = 108). Thematic analysis was performed on 363 narrative interviews to identify R&S themes and content related to decision-making.

**Results:**

Parents from 13 cases reported R&S directly influenced decision-making. Most HCPs were unaware of this influence. Fifteen R&S themes appeared in parent and HCP transcripts. Themes most often associated with decision-making were *Hope & Faith*, *God is in Control*, *Miracles*, and *Prayer*. Despite instability in the child’s condition, these themes remained consistently relevant across the trajectory of illness. R&S influenced decisions about treatment initiation, procedures, and life-sustaining therapy, but the variance in effect of R&S on parents’ choices ultimately depended upon other medical & non-medical factors.

**Conclusions:**

Parents consider R&S fundamental to decision-making, but apply R&S concepts in vague ways, suggesting R&S impact *how* decisions are made more than *what* decisions are made. Lack of clarity in parental expressions of R&S does not necessarily indicate insincerity or underestimation of the seriousness of the child’s prognosis; R&S can be applied to decision-making in both functional and dysfunctional ways. We present three models of how religious and spiritual vagueness functions in parental decision-making and suggest clinical applications.

**Electronic supplementary material:**

The online version of this article (10.1186/s12904-018-0360-y) contains supplementary material, which is available to authorized users.

## Background

Advances in life-sustaining technology for seriously-ill children have opened the door to new complexities in decision-making for parents and health care providers (HCPs) [1]. Within a short timeframe, physically and emotionally exhausted parents must learn about their child’s condition, inform themselves of treatment options, and weigh risks and benefits [2–4]. Families rely on HCPs to understand decisions they face [3, 5, 6], and many families also rely on non-medical sources of support, like religion and spirituality (R&S) [2, 3, 7–15]. Parents often rank R&S among the most important decision-making factors, especially for high-stakes decisions [13, 16–19]. Most HCPs recognize R&S as part of pediatric palliative care [20], but many feel unqualified to offer guidance in this realm [21–24]. Use of palliative and pastoral care services is increasing [25–28], but R&S-related conflict, miscommunication, and lack of understanding between parents and HCPs persist [29–32]. Examining both parent and HCP perspectives on the role of R&S in decision-making may provide direction to HCPs.

Studies examining R&S in pediatric palliative care have focused on their role in parental coping and usually rely on single time-points or retrospective reports. Prior cross-sectional research has identified R&S themes but has failed to clarify how R&S influence decision-making or determine particular effects of R&S factors. Longitudinal trajectory designs are particularly powerful for studying how R&S factors impact parents as they respond to the trajectory of infant chronic critical illness with its characteristic unpredictability and dynamic changes [33, 34]. Parents’ descriptions of how R&S influence their decisions can be elusive and rich in aphorisms [9], which HCPs may see as insincerity, poor understanding of prognosis, or denial [35, 36]. R&S worldviews provide context for parents to interpret circumstances and make decisions. The complexity of this context requires in-depth, longitudinal exploration of parent and HCP perspectives on R&S-guided decision-making. The aim of this study is to illuminate the influence of R&S on parental decision-making and explore how providers interact with parents for whom R&S are important.

## Methods

### Study design, participants, and setting

A qualitative descriptive design was used to explore decision-making trajectories for infants with complex life-threatening conditions (CLTCs) (R01NR-010548-01A1). Interviews with parents and HCPs from a subset of cases underwent analysis of R&S content. Each study case included an infant, at least one parent, and at least three HCP (physicians, nurses, nurse practitioners (NPs), and social workers). Data from each case consisted of a longitudinal series of one-on-one interviews, field notes, questionnaires, and medical chart data (from both inpatient and outpatient encounters) collected across the infants’ illness trajectory. Interviews ranged in length from 30 to 90 min with the majority of parent interviews lasting longer than one hour. Infants receiving care at a southeastern U.S. academic medical center were eligible for the principal study if they had one of three kinds of CLTCs: extreme prematurity (< 26 weeks-gestation), complex congenital heart diseases, or genetic disorders requiring hematopoietic stem cell transplant (HSCT). Purposive sampling occurred from 2008 to 2011 to enroll a population of cases (*n* = 33) representative of the clinical population. A subset of cases was sampled for R&S analysis if the study entry transcript review revealed R&S content. Thematic saturation was achieved after 13 cases. Three additional cases were analyzed to validate saturation (*n* = 16) [37]. All transcripts from these cases were included in data analysis. The 16 cases analyzed for R&S content included 28 parents and 108 HCPs. Tables 1, 2, and 3 show sample characteristics. Additional file 1: Table S1 includes a more detailed breakdown of the participants involved in each case. Notably, 27 of the 28 parent participants were Christian (protestant, Catholic, or unspecified). Of the remaining 17 cases not analyzed, some included R&S content thematically similar to that of the included cases, while others contained little to no R&S content. The sample size was small enough to allow intensive study of 363 interviews, while permitting variation in demographics and R&S worldview.

**Table 1:** Demographics of parent participants

Variable	Number (%)
Total Participants	28
Sex	–
Male	12 (43%)
Female	16 (57%)
Ethnicity	–
Caucasian	11 (39%)
Hispanic	5 (18%)
African American	10 (36%)
Native American	2 (7%)
Average Age (range, SD)	32 (21–46, 6.4)
Average Years of Education (range, SD)	14 (7–18, 2.5)
Married	–
Yes	23 (82%)
No	5 (18%)
Religious Preference^a^	–
Christian	27 (96%)
Other	1 (4%)
Income	–
< $15,000	3 (11%)
$15,000–$25,000	4 (14%)
$26,000–$50,000	7 (25%)
$51,000–$75,000	6 (21%)
$76,000–$100,000	5 (18%)
> $100,000	3 (11%)

^a^Demographic survey allowed participants to select from 5 options: Christian, Jewish, Muslim, Hindu, and Other (with option to specify a particular religion). “Christian” is broadly defined to include any individual who self-identified as “Christian” based on their beliefs, denomination, or sect. The participant who selected a religion of “Other” did not specify a particular religion but described themselves as “spiritual” when interviewed

**Table 2:** Demographics of infant participants

Variable	Number of Participants (%)	Average Age at Study Entry (range, SD)	% Living at Study Exit
Infant Diagnosis			
Complex congenital heart disease	5 (30%)	22 days (1–61, 27)	40%
Genetic/metabolic disease/HSCT	7 (40%)	11 months (3–21, 6)	71%
Extreme prematurity	5 (30%)	0 days (0–2, 1)	40%
Total	17^a^	148 days	53%

^a^One case included twins

**Table 3:** Demographics of HCP participants

Variable	Number (%)
Total Participants	108
Clinical Position	–
Physician - Attending	30 (27%)
Physician - Fellow	5 (5%)
Nurse Practitioner	25 (23%)
Nurse	27 (23%)
Social Worker	22 (21%)
Sex	–
Male	31 (29%)
Female	77 (71%)
Religious Preference	*107*
Christian	79 (74%)
Jewish	7 (7%)
Hindu	8 (7%)
Other	13 (12%)
Average Years of Experience	
Total Clinical Experience (range, SD)	12 (0–30, 9.3)
Experience in Current Clinical Setting or Specialty, i.e. NICU, BMT, etc. (range, SD)	8.3 (0–30, 8.7)

### Data collection

Enrollment began at the decision to transplant for the HSCT group, and at birth or diagnosis for the other diagnostic groups. Cases were followed throughout inpatient treatment and at follow-up clinic visits. Of the 16 cases sampled, the shortest case study lasted 8 days and the longest lasted 531 days (median = 380 days, mean = 324 days, SD = 174 days). Assistants trained in narrative interview techniques conducted one-on-one interviews (in person or by phone) with each parent and current HCP. Any HCP caring for the child at the time of the interview was eligible to participate. In cases where a single HCP cared for multiple patients in the study, they were asked to speak about these cases separately. Interviews were conducted first at study entry and subsequently at monthly intervals, within one week of life-threatening events or treatment changes, within two weeks of discharge, and 12 months following enrollment. For cases where the child died during the study, parents were interviewed within six weeks after the child’s death. Narrative interviews allowed parents to share their experience of the infant’s illness, treatment, and bereavement (when indicated). The interview guide included questions about R&S, and interviewers probed further when interviewees affirmed a role for faith, religion, or spirituality in their lives (see Table 4 for sample questions). When parents denied any role for R&S in their lives or decision-making, they were not probed further during that interview, but interviewers continued to ask about R&S in subsequent interviews. Interviews were electronically transcribed. Names of people and places were replaced with pseudonyms. Ultimately, 61% of parent interviews were conducted while patients were admitted to the hospital, 37% were conducted while patients were receiving outpatient treatment, and 2% were conducted after the child had died (bereavement interviews).

**Table 4:** Sample interview questions

Participant Type	Sample Questions
Parent	Interview Guide: *“Has your spirituality or belief in God influenced your decision-making?”* Probe: *“Tell me more about your faith, where that comes from, and how that plays a role in your life.”* Follow-up: *“Where are you with your faith, and are you continuing to feel that way or do you feel differently?” or “Does your faith have a different place after going through this process?”*
Health Care Provider	Interview Guide: *“What factors do you think influenced the parents’ decisions (for example, spirituality, family, past experience)?” or “Are you aware of anything that influence the parents regarding their faith?”*

### Data analysis

Analysis of R&S content was conducted on interview transcripts using content analysis techniques described by Hsieh and Shannon (2005) [38]. Goals were to (1) identify R&S themes and characterize the relationship of each theme to decision-making, and (2) assess differences between parents and HCP perspectives on R&S. Text-based qualitative analysis software (NVivo, QRS) was used to view, organize, and apply thematic codes to data. Three authors reviewed all transcripts. The first author then proceeded to identify and code any statement with reference to R&S, using conventional content analysis [38]. To validate coding of all relevant content, a text query for R&S-associated words was performed, using words frequently found around R&S content, or identified in prior studies (using summative and directed content analysis techniques, respectively) [38]. The codes and example statements developed by the first author were then presented to all authors and a consensus process was used to clarify codes and definitions. Inductively-derived themes developed by all authors were then applied to R&S-coded content. Similar codes were grouped into categories. Content within each thematic code was re-examined to define themes and sub-themes. After completion of thematic coding, R&S-coded content that referenced decision-making was also identified and coded as such. During coding and categorization, co-authors met to discuss and validate the coding framework. Finally, content was reviewed to characterize the relevance of each theme to decision-making, identify changes over time, and compare R&S content derived from HCP and parent interviews. While identifying changes in R&S content over time, attention was paid to the particularities of each case, including the child’s diagnosis, current condition, family composition, and reported interactions with HCPs. In addition to data source triangulation (collection of data from parents and multiple types of HCPs), method triangulation was achieved by using questionnaires and medical chart data to corroborate and clarify interview reports [37].

## Results

R&S references appeared in every case, and parents from 15 cases stated religion was important to them. Parents from 13 cases said R&S influenced their decision-making. In 12 cases, parents said R&S influenced specific major decisions, including treatment initiation, choice of hospital, and prioritizing goals of care (Table 5). R&S content was present throughout the illness trajectory, though not necessarily discussed during every interview. The role R&S played in decision-making varied widely within and between cases. Parents relied on both R&S factors and medical data, including the physician’s recommendation, and usually did not see the two in conflict (e.g. “Faith and belief in God…it’s part of that whole analytical process and I don’t separate it”). In some situations, parents spoke of a conflict or contrast between medicine and faith (e.g. “They say they understand [that] we believe in our faith, but they still [talk] about medical terms [and] science.”) Notably, of the 24 physicians involved in cases where R&S influenced major decisions, only four (from three cases) reported awareness of the influence R&S had on decision-making. In one case where R&S played a role in parents’ refusal to withdraw care, one physician explained that “the parents were very reluctant to [withdraw care] because of their religious beliefs,” while another physician who cared for the family concurrently said, “I didn’t recognize [faith] as something that was driving them.” Physicians’ awareness of parental R&S usually came from observation or second-hand reports (e.g. seeing parents pray or knowing a chaplain visited), where as many nurses, NPs and social workers had personally discussed R&S or prayed with families. While a detailed report of the differences in R&S-themed quotes among different types of HCPs is beyond the scope of this paper, representative HCP quotes are provided in Additional file 1: Table S2 for reference.

**Table 5:** How religion and spirituality influenced major decisions. Description of R&S influence is summarized from parent reports, unless otherwise stated. Themes are underlined

Major Decision	Cases Represented	Description of R&S Influence
Locus of Care	4	- *Prayed* about choosing a hospital. - Received signs or heard *God’s voice* indicating a certain hospital. - *Blessings* and signs confirmed these decisions. - Expected to see *miracles* at certain hospitals. - Required *faith* and trust in *God* to relocate during treatment for a higher level of care, or to transfer to a local hospital with a lower level of care. - Knew *God* would be with them wherever they went, and would provide.
Treatment Initiation	4	- *Prayed* about initiating or choosing a treatment plan. - Delayed initiation while *praying* and waiting for peace from *God*. - Able to initiate high-risk treatment because *God* would be present.
Life-Sustaining Therapy (Continue vs. Withdraw)	8	- Continued therapy, maintaining *hope* and *faith* in *God*, or because it should be God’s decision. - *Clergy* prohibited removal of endotracheal tube (per HCP). - Withdrew therapy, reassured that *God was in control*, there is *life after death*, and the child is no longer *suffering*.

Fifteen R&S themes were identified and organized into four categories: Values/Beliefs, Practices, People, and Emotions. In Table 6 we have provided detailed theme definitions, subthemes, and exemplary quotes that may assist readers in judging the dependability and transferability of our findings. Each theme was identified in at least one parent transcript, but not all themes were represented in HCP transcripts. For each case, the prominent themes were stable over time. Themes most often cited as influencing parents’ decisions were *Hope & Faith*, *God is in Control*, *Miracles*, and *Prayer*. Twelve themes were associated with descriptions of decision-making (overall process or specific decision). Below, we describe the major findings within each theme, focusing on applications to decision-making. Due to the interrelatedness of the themes, descriptions are not entirely discrete, but we aim to depict the essentials of each theme and the relationships between themes, as observed in interview transcripts.

**Table 6:** Theme definitions, subthemes, and exemplary quotes. themes associated with decision-making are marked with an asterisk (*)

Theme	Definition	Exemplary Quotes
- Sub-themes	References are bracketed.	MD = physician, NP = nurse practitioner, RN = nurse, SW = social worker
I. Values & Beliefs	*R&S principles and convictions that guide a person’s behavior, choices, and interpretation of events.*	
1. Hope & Faith* - *Faith in God* *- Faith in Medicine* *- Faith in Self* *- Optimism* *- Commitment to Hope/Faith*	*Theological virtues in the Christian tradition. Hope is an expression of trust, positivity, or desire independent of naturalistic justification. Faith may be a synonym for hope, a synonym for trust, or acceptance of a belief system (*i.e. *Christian gospels)* [67]*.*	*Parent*: “For me, having faith means that everything will turn out okay. And therefore I don’t really stress. I think, and re-think decisions, but I don’t get stressed out about decisions, because my faith tells me everything will be okay.” *MD*: “Deep religious belief is difficult sometimes. It’s great sometimes, when it gives you hope and when we still have hope and there are problems and you want to just keep them going and they can just dive into that. It’s a real great grounding for those things where you still have a chance. It’s a 5% chance, but there’s still 5%, and that’s great ‘cause it makes it a lot easier, so that’s the upside. Now the downside of it is when there’s zero [percent chance] and they want you to do things, and in your best judgment that’s just not good for anybody.”
2. God is in Control* *- In God’s hands* *- God knows (best)* *- Man is not in control* *- God’s plan or will* *- God decides* *- God provides* *- God’s grace or mercy*	*Expressed belief that God is good and has supreme power, over human beings, worldly situations, and “natural” physical forces.*	*Parent*: “He’s in control of everything and there’s nothing I as a person can’t do that [God’s] not in control of. So if He wants things to get better, it will get better, and if He doesn’t want it to get better, it won’t be.” *MD*: “The parents were very reluctant to [withdraw care] because of their religious beliefs. They were very strongly believing, quote-unquote, ‘We’ll leave it in God’s hands,’ despite the fact that we were using medicines to keep things open, and I think that was a very difficult problem for the staff versus the parents.”
3. Voice/Presence of God* *- God speaking* *- Divine guidance* *- Signs* *- God is with us*	*Belief that God communicates with people by audible voice, external signs, or internal feelings. Also, a sense that God is near.*	*Parent*: “I took a day in my room and I was like, ‘God, I’m not leaving until you tell me what to do. I’m not moving from this spot until you tell me in my heart what I’m supposed to do.’” *SW*: “Mom kept saying she was praying about it, and she was waiting for an answer, but it wasn’t quite coming, and then finally, she did. She said, ‘Okay, I’m ready. I’m gonna sign [the BMT consent paperwork].’ And she said, ‘Maybe this is my sign—that everybody else is feeling positive about this.’”
4. Miracle* *- Unexplained healing* *- Hope for a miracle* *- Awe or wonder* *- Improbability* *- Miracle babies*	*Event that transcends the Laws of Nature by divine intervention* [39]*.*	*Parent*: “[My faith] plays a tremendous part into my decision-making, and I believe God can do anything anytime he gets ready. I do believe in miracles.” *MD*: “I told them there were no chances for miracles, there was no chances for survival, and there were no chances for normalcy at all.” *RN*: “The parents looked [at] it from more of a religious standpoint. She was born, she’s living, this is a miracle, we’re going to let God take its course. We don’t want to be the ones to decide whether to remove her support.”
5. Meaning of Suffering* *- Suffering has a purpose* *- Good from suffering* *- Asking “Why?”*	*Ascribing purpose to pain, disease, hardship, and death, or questioning whether there is a purpose, and whether it is just.*	*Parent*: “I understand that God has something—really, really, really has to have something—very nice for me, because after all the things that we’ve been going through (and it keeps on going), you really have to see the bright side.” *MD*: “We all collectively screwed up including the parents by making this happen. The nurses looking and saying, ‘That’s suffering.’ The parent saying, ‘That’s not suffering.’ It’s kind of an interesting observation. I don’t know which to do with it. It’s like looking at people’s lives—a person with CP—and saying it’s so bad that the person would rather not be alive.”
6. Meaning of Life* *- Sanctity of life* *- Brevity of life* *- Purpose of a life*	*Ascribing purpose or value to human life, in general or for a particular person.*	*Parent*: “I truly believe everything in your life is meant for good and it’s just a part of the journey, and I kept saying, ‘God, I want to be able to see the beauty in this process.’”
7. Meaning of Death* *- Afterlife* *- Superstition*	*Religious doctrine or spiritual convictions about death and afterlife, as well as expressed feelings and fears.*	*Parent*: “I look at it as, she’s in a better place and she’s one of God’s little angels.” *RN*: “A lot of them do suffer greatly before they die, and it’s nice if you can believe that they’re out of suffering and in a much better place, so there was a reason for their life and a reason for their death…It’s nice for the parents, too, when honestly they have some religion to fall back on because then they feel that there is also a purpose and meaning and it can help them move on as opposed to being bitter.”
II. Practices		
8. Prayer* *- For good outcomes* *- For strength* *- To saints* *- About decisions*	*Individual or communal petition to a divine being, saint, or spirit.*	*Parent*: “I prayed about [my decision]. I just turned it over to God, because He’s in charge of all of this anyway. I guess that’s how I was comfortable with my decision, because I had prayed about it.” *NP*: “She prayed about [the decision] and I think that probably, maybe, helped her in some way, on some level make her decision.” *RN*: “I’d give mom the little syringe of blood and she’d say a little prayer before I put it into the little container and the little lab rack: a ‘grow, cells, grow’ prayer”
9. Scripture	*Reading, reference, or belief in a religious text.*	*Parent*: “When I feel like I need some strength to get out of this bed to go on, to move, to help my day, I’ll grab my Bible.”
10. Baptism	*Christian rite in which a person is united with Christ in death and resurrection through either submersion in, or sprinkling with water.*	*Parent*: “Right after I got off the phone with the wife, and knew absolutely nothing, ‘cause she was crying so much, I went back to my room, got on my computer, and sent a message off to the assistant pastor at our church and said, ‘Go baptize [our daughter].’” *RN*: “If they don’t want to bring in a church person or religious [person], they can even have one of the nurses do it, ‘cause a couple of the nurses who are authorized to baptize.”
III. People & Community		
11. Faith Community *- Friends* *- Church service*	*People bonded by shared R&S, who may pray, worship, or serve together.*	*Parent*: “I went to [the] church that we had become a part of here. They came over with tons of food, which was just a huge blessing, because they said they didn’t want us to have to worry about trying to cook or any of that for a week.”
12. Faith Leaders* *- Pastors* *- Priests* *- Hospital chaplains* *- Elders & deacons*	*Individuals with religious or spiritual authority, who may provide spiritual teaching, guidance, and counseling, and support or perform sacred rituals.*	*Parent*: “We made a good decision by bringing [the chaplain] in. It’s one of those conversations you have to have.” *MD*: “To [the chaplain], I think they were more open in how they said that they understood that the baby was going to die and they were okay with that—well, not *okay*, but you know. They accepted that it was going to die, but their pastor or their religion would not allow them to pull the ET tube.” *RN*: “They saw [the chaplain] and [Mom] was like, ‘No, no, no. He can’t come in. He can’t come! No, no.’ And I’m speculating, but I think to a lot of people [a chaplain] is indicative of ‘If I let you in, then [my child’s] going to die.’” *RN*: “Especially as children die, almost everyone wants a chaplain or they want some spiritual support, even if they just use it for the short-term. I don’t think I’ve ever had anyone who hasn’t requested a chaplain, honestly, at some point.”
IV. Emotions	*Emotional reactions to life experiences, grounded in R&S worldviews.*	
13. Gratitude* *- Blessings*	*Sense of appreciation, general or specific.*	*Parent*: “That’s the process for me—recognizing what actually happened, then what good happened in it, what can I pull from it? And then I go into gratitude.”
14. Strength & Growth* *- Stronger faith* *- Grow in love*	*Sense of positive personal development, mainly in virtues of faith, hope, and love.*	*Parent*: “I was able to walk away and say, ‘Okay, God, I get it now. My capacity for compassion and love and joy has multiplied more than I could ever imagine.’”
15. Anger (at God)	*Animosity, often due to suffering or unanswered prayer.*	*Parent*: “I’m like, ‘You’re not in my situation. You don’t understand. I can be mad at God if I want to be.’ But then it brought me closer to Him.”

### I. Values and beliefs

#### *Faith & Hope* (Theme 1)

Every parent referenced the related concepts of faith and hope. “Faith” and “hope” are sometimes synonymous, but faith can also mean “trust” or “belief system,” and hope can mean “expectation” or “desire.” References were fittingly diverse, but unified by an optimistic tone and religious connotation. Parents believed faith was integral to decision-making in that it gave them confidence in decisions, guarded against regret, and aided joint decision-making with their spouse. Having faith that God would provide for the family (e.g. job, finances, community, etc.) also allowed parents to focus on the decisions before them. After making a decision, it was important to maintain hope for a good outcome. When asked to rate their hope, parents almost uniformly were either “very” or “extremely” hopeful, even after life-threatening events, receiving bad news, and when prognosis was poor.

Fear of disappointment tempered parents’ hope, but longitudinal analysis revealed that no parent reported that they had lost faith in God or lost hope that their child would get better. As decisions became more complicated or consequential (e.g. new devices, goals-of-care, end-of-life, etc.), parents spoke more emphatically about the importance of maintaining hope and faith. Many parents spoke of “faith” as a parental responsibility, especially as the child’s condition was worsening. Parents implied that maintaining faith could impact their child’s outcome (e.g. “If you don’t have faith, maybe everything goes down.”).

HCPs had mixed feelings about parental hope and faith; faith kept parents hopeful enough to be involved and endure stress but became problematic when cure was no longer possible, from a medical standpoint. While parental hope was stable over time, HCP hope fluctuated with the child’s medical status or prognosis. Over time, many HCPs began to worry that faith-based hope was allowing parents to disregard medical evidence when making decisions. As one NP explained, “I think it’s perfectly fine for you to hope to the very last second that this baby’s going to make it, but we’re going to talk about reality right now, we’re going to talk about what the likelihood is.”

#### God (Themes 2–3)

Parents spoke about the nature and presence of God. Because almost all parent participants were Christian, parents’ depictions of God were similar and consistent with the God of Christianity—an all-powerful, all-knowing, and perfectly good divine being who created humans and whose spirit is present and active in the world. All mothers and most fathers emphasized the belief that *God is in Control* (Theme 2). At times, this belief empowered parents to make decisions (citing that God is in control of their decisions); at times, it motivated parents to abstain from making decisions (citing that God should decide).

Many parents said it was important to “give over” control to God—to put “everything in God’s hands.” Surrendering control to God freed parents from the burden to control chaotic situations themselves. They admitted this was not easy or straightforward and wanted to remain engaged in their child’s care. They did not expect HCPs to surrender control to God but seemed pleased when physicians acknowledged a higher authority. In many cases, HCPs believed sacrificing control should mean letting “nature take its course.” Some parents agreed, while others believed it meant doing “everything” possible to keep the child alive (i.e. continuing life-sustaining therapy) until God made a final decision. Over time, life-threatening events and medical instability did not negate parents’ assertions that God was in control; rather, parents were more likely to bring up this belief in difficult circumstances. Believing God was in control assured parents that the situation was not as chaotic as it seemed. One mother who had lost a child prior to the study said the experience taught her God was always in control, “so if he wants things to get better, it will and if he doesn’t want to get better it won’t,” but this allows her to focus on each new challenge as it comes.

The *Presence or Voice of God* (Theme 3) was another prominent theme. A few parents recounted listening to the voice of God in prayer. One mother vividly recalled, “there are certain moments in my life that I knew that God was speaking, like when he told me [my husband] was my husband. I knew, I just knew. And the day that I was journaling and writing, I said, ‘The GJ [gastrojejunostomy] is what she’s supposed to have.” Most parents had a more general sense of God’s presence and support—“I know there’s a God and he was there in that surgery room. I know that for sure. And he’s still there with my little girl right now.” One father felt God led him to the decision to take a job closer to his child’s hospital, stating, “God didn’t come out of the sky and say, ‘you must move’ … I just felt that it was the right thing to do.” Many said they could not have endured their circumstances or made decisions without God’s presence.

#### *Miracles/Divine Intervention* (Theme 4)

Nearly half of parents spoke about miracles or alluded to divine intervention. Belief in miracles was related to beliefs about God and influenced decisions in similar ways. If God is in control, then God can intervene in the world and bring about events that defy medical explanation [39]. Believing in miracles sometimes pushed parents to pursue aggressive treatment, and other times allowed parents to de-escalate aggressive care. Three sets of parents insisted their children maintain full code status, because they believed in miracles. In one of these cases, the physician tried to convince the parents their child was dying by drawing diagrams and explaining pathophysiology, but the parents maintained that “man” was not in control; they knew from experience that God performs miracles. These parents did not feel physicians understood their beliefs. After a rocky course, they did decide to withdraw care, but never gave up their belief in miracles, explaining, “We don’t blame it on God, we just believe that God knew…He knew what he was doing.” Conversely, another parent demanded discontinuation of anti-hypertensive therapy, believing that if the child became hypertensive and had a stroke, God would “take care of it.”

Parents and HCPs used the term, “miracle babies,” to describe children who had received devastating prognoses but ultimately exceeded medical expectations. To parents, if God miraculously brought their child into the world, he would miraculously keep them alive. Thus, they were less likely to accept poor prognoses or “give up” hope. HCPs used the term, “miracle,” more reluctantly (e.g. “I hate to use the term miracle baby, but…”). Some HCPs said their experience with medical miracles made them less confident in their ability to “predict the future” and more cautious when communicating poor prognosis.

#### *Meaning of Suffering* (Theme 5)

The belief that God is perfectly good affected how parents interpreted suffering. Either God predetermined a purpose for suffering, or he could bring good things from suffering. While some parents had ideas about why God might allow their child to suffer (e.g. long-term benefits, personal growth, etc.), others were just comforted that God “knows best” and “has a plan.” This theme was especially prominent in bereavement interviews, where parents often said God must have known something they did not. The issue of suffering seemed to be the greatest point of contention between HCPs and parents. HCPs believed suffering was only allowable when necessary to prolong a life of good quality. They felt parents used R&S beliefs to “rationalize” the infant’s short-term suffering. In one case, a physician stated that the parents “just [didn’t] care” that the infant was suffering.

Most parents also acknowledged doubting whether suffering had meaning (“asking, ‘why?’”). While some parents endured long periods of wrestling with questions, but none reported a change in their faith or beliefs based on this questioning. One mother noted that questioning and doubt was a necessary part of the process, saying she had to get out the “kicking and screaming” and “find the gifts.” Data did not support a finding that parents who lost children (either during or prior to the study) had greater clarity on the meaning of suffering than parents who did not experience loss, but these parents did express less distress over these questions. Many questioning parents relied on the support of spouses, chaplains, and religious friends, but no one explained what these people did or said that was helpful.

#### *Life & Death* (Themes 6–7)

Parents generally believed that there was life after death, and that the afterlife was a good, peaceful place. For many parents, this alleviated the fear of death. For parents who had previously lost children, the ability to maintain a connection with the child after death helped them to see death as “not that bad.” For cases where the child died during the study, parents’ beliefs about the afterlife did not appear to change significantly, based on bereavement interviews. They spoke of their child’s enduring spiritual presence as near and interactive, as well as distant or “in heaven.”

Parents acknowledged that life is short and sacred, and many believed each life has a purpose. When parents believed they were “meant to be” their child’s parents, they were empowered to trust their instincts about what was best for the child.

### II. Practices

R&S practices included rituals or actions that call upon or respond to a higher power. Examples from this study include *Prayer* (Theme 8), *Reading the Bible* (Theme 9), *Baptism* (Theme 10), and anointing of the sick. Prayer was by far the most prominent.

#### *Prayer* (Theme 8)

Prayers were directed toward either God or individual saints, and sometimes involved the use of rosaries, relics, or oils. Twenty-seven of the 28 parents attested to praying at some point, and most prayed regularly. Parents believed prayers made a difference in their child’s condition. They appreciated when HCPs (usually nurses) prayed with them. The pattern of prayer across the illness trajectory was unique to every case. While some parents prayed consistently throughout the time their child was sick, others described “prayer exhaustion” around the middle of treatment. In one case, parents who did not pray started requesting prayers from others when they learned their child was dying—specifically, prayers the child would “go to heaven.”

Prayer also guided decision-making. In four cases, it played a large role in parents’ decisions, including decisions about treatment initiation, choice of hospital, medical procedures, relocation, resuscitation orders, and withdrawal of life-sustaining therapy. Two parents from different cases reported praying over every decision throughout the illness trajectory and believed God sometimes directed them toward specific choices. Parents did not always state the way in which prayer guided these decisions but were clear that prayer engendered peace and confidence in their choices. In other cases, prayer was an accessory to decision-making. After making a decision, some parents prayed they had made the right decision, or that good things would come out of the decision. Although prayer did not always instruct decisions, many parents said they could not have made decisions without prayer.

### III. People

There is a significant social component to R&S, consisting of faith communities, faith leaders, and communal rituals, all of which were important to parents in this study.

#### *Communities* (Theme 11)

Christian friends and church communities supported families in this study through prayer, fundraising, meal preparation, visiting, child care, and other day-to-day tasks. These communities did not directly impact decision-making, although one family did suggest support from their church community reinforced their decision to leave the hospital and care for their child at home.

#### *Faith Leaders* (Theme 12)

Pastors, priests, and hospital chaplains provided spiritual and emotional support. In one case, HCPs reported a family’s pastor prohibited endotracheal tube removal, and they abided by that condition while de-escalating care in other ways. Otherwise, faith leaders did not directly influence decisions. Families appreciated hospital chaplains, even if they already had a pastor or priest. HCPs spoke highly of chaplains and routinely involved the pastoral care team when a family seemed religious or a child was in critical condition.

### IV. Emotions

The following themes are feelings directed at a higher power (e.g. anger or gratitude toward God) or inspired by R&S beliefs (e.g. growth as part of God’s plan).

#### *Gratitude* (Theme 13)

All parents referenced feeling grateful and blessed. They were especially grateful for the support and health care they received from hospital staff, but were also thankful to God for their child’s life and for any improvements in their child’s condition. Paradoxically, expressions of gratitude were as frequent when the child’s condition was declining as when the child was improving or stable. Certain blessings validated decisions parents had made.

#### *Growth* (Theme 14)

Some parents saw the experience of their child’s illness and treatment as a journey of positive spiritual growth. Many noted how God helped them grow in their decision-making abilities. In final interviews, most parents felt their faith was stronger after the experience, even if they experienced periods of anger or doubt.

#### *Anger* (Theme 15)

Parents admitted sometimes being angry with God. Parents became frustrated that their prayers weren’t being answered, or that they kept getting their hopes up only to be disappointed. Nevertheless, parents’ anger at God always subsided. None of the parents who participated in bereavement interviews after the child’s death expressed anger or resentment toward God. One parent did express continued resentment toward physicians for apparently abandoning hope for their child.

## Discussion

Our results reinforce R&S as a source of personal comfort and guidance to many parents of seriously ill children, and expound on prior descriptions of the role R&S play in parental decision-making. We found R&S themes similar to those discovered in previous studies [13, 16–19], and a longitudinal perspective gave us a deeper look into how aspects of R&S influence decision-making. Across the illness trajectory, parents applied vague concepts, rather than specific or instructive doctrines, to make decisions. Parents’ expressions of these R&S factors were generally steady over the course of the child’s illness and reportedly affected different types of decisions in different ways at different times. While HCPs appreciated some benefits of parental R&S, they were frustrated when parents applied R&S to decision-making in seemingly contradictory or unclear ways. The relative “vagueness” of the way parents apply R&S may have an important role in the way such themes function in parental decision-making and coping.

We want to delve deeper into why R&S matter to parents when they are making decisions, and explore the role vagueness plays in their ability to hold it treasured. In the following discussion, we further describe religious and spiritual vagueness, and present theoretical models for its role in decision-making. We conclude with applications for clinical practice and future research.

### Explaining R&S vagueness

We expected discrete cause-and-effect relationships between some R&S factors and downstream decisions, but we discovered a more nuanced influence. Our results suggest R&S may affect *how* decisions are made more than *what* the decisions are. Parents’ beliefs did not change over time, but the effect of a single belief on ultimate choices differed in different circumstances. For example, parents whose belief in “God’s plan” initially motivated a preference for aggressive life-sustaining therapy later affirmed that belief in God’s plan gave them peace about withdrawing care. Parents used vague terms to explain how faith, hope, and trust in God were important (sometimes indispensible) to their decision-making process.

We use the term “vague” to describe a concept that is imprecise, sometimes inconsistent in its expression and application, and not dependent on a detailed or mechanistic understanding [40–43]. As practitioners of health sciences, HCPs seek precision and make decisions based on probabilities [44–47]. Conversely, R&S are often characterized by mystery, symbol, and complex belief or philosophy. Such features allow for freedom and possibility amidst a reality that is beyond human capacity to fully grasp, making R&S increasingly vital as medical probabilities become less favorable [42, 48, 49].

The variation we observed in HCPs’ attitudes toward R&S—from appreciative, to apathetic, to frustrated—suggests the presence of biases about R&S that may impact their practice [8, 50]. .A 2010 survey found that U.S. physicians are more accommodating of patient appeals to instructive doctrine compared to “religious” hopes [51]. Similarly, we found that HCPs sometimes interpret the vagueness and apparent paradox of parental expressions of R&S as an attempt to legitimize emotion-driven desires (intentional or unintentional). Consistent with prior studies [52, 53], HCPs in our study were usually unaware R&S was influencing decision-making. The persistence of R&S-related conflict and miscommunication between parents and HCPs indicates the need for a new framework of understanding the complex role of R&S in parental decision-making [29, 31, 32, 54, 55].

### Models to explain the amorphous role of religion & spirituality in decision-making

R&S influence decisions differently depending on many factors, but there are no theoretical frameworks to describe these different influences. Here, we propose three models to describe the role of R&S in complex health-related decision-making for parents. These models arose from recurring narrative themes in the cases we examined (Table 7). In constructing these models, particular attention was paid to ways in which a single belief or principle could influence decision-making in seemingly contradictory ways. Each model is distinct, but they are all founded on recurring statements parents made about the distinction between God and man, the belief that God works in the world, and the acknowledgement of uncertainty. Thus, the models are not mutually exclusive. R&S probably function at different levels simultaneously in each case and each model may be applied in functional and dysfunctional ways.

**Table 7:** Quotes & narrative arcs supporting proposed models for influence of religion & spirituality in decision-making

Model	Exemplary Quotes & Narrative Arcs
1. Means of Confronting Difficult Decisions (without abandoning hope)	A mother took significant risks to relocate so that her child could start an aggressive treatment regimen. She believes she was only able to make this decision because she had faith that God would provide for her and her child throughout the many hardships and uncertainties. *“I think and rethink decisions, but I don’t get stressed out about decisions, because my faith tells me that everything will be okay when it all comes out in the wash. It may not be from one day to the next, but if you have faith, you know that it will.” (Mother, regarding approach to decision-making in general)*
2. Means of Delaying Acceptance of Harsh Realities	Parents of a child with a fatal prognosis refused to discuss the possibility of death, comfort care, or negative prognosis with physicians, citing their commitment to hope and their belief in miraculous healing through prayer. This refusal persisted despite numerous attempts by physicians to inform and educate the parents about the child’s condition. *“They just kept speaking death over my child all the time. We had already put that in the back of our mind, in case that worst-case scenario did happen. You know, we would have to accept it.” (Father, regarding refusal to have goals-of-care discussion)*
3. Foundation for Trust—in God, Physicians, and Self	A mother relied on prayer for decision-making throughout the child’s illness, slightly delaying treatment initiation and certain procedures, citing belief that God is in control, that God equips her to make decisions, and that God is working through physicians. *“I just turned it over to God, because he’s in charge of all of this anyway … and there was only so much my mind could absorb and … I knew that my final decision was good because I had prayed about it.” (Mother, regarding decision to undergo a procedure)*

#### #1: Means of confronting difficult decisions (without abandoning hope)

For the parents in our study, one of the greatest decision-making challenges was accepting uncertainty and harsh possibilities they never imagined and could not have prepared for. R&S allowed parents to acknowledge harsh possibilities while holding on to hope for a good outcome. Faith and medical data played complementary roles in decision-making; data informed their choice, but faith, prayer, and belief in God’s control provided strength to consider difficult possibilities and accept that uncertainty is inevitable.

This observation is consistent with the understanding that the practice of medicine focuses on solving mystery with data, while the practices of R&S provide ways to embrace mystery by allowing for unseen realities [45, 48]. Uncertainty need not be resolved before committing to a decision; rather, it creates an opportunity to hope and have faith. While this role for R&S is typically relegated to the category of “coping,” [9, 10, 15, 56] the ability to accept harsh realities and possibilities is fundamental to decision-making as well [57]. Parents prayed to express questions about the meaning of suffering, difficult choices, and the uncertain future. Bringing concerns to God allowed parents to find acceptance and confront the next challenge.

Many parents are comforted by the beliefs that God is in control, knows best, and has a plan, *no matter what the outcome is*. One mother explained, “faith allows me to take a hands-off approach and just trust this process and trust that [my daughter] is having the experience that she is supposed to have and that it’s the purpose of her life. And I don’t want to take that away from her.” Believing that God is in control both sustains hope that the child will be healed and offers reassurance that regardless of the outcome, everything will be okay. Therefore, parents can make decisions with humility—their decision may not be perfect, but it is not the end-all-be-all. As another mother said, “I know God is in control and he knows. He’s the one that has never failed, so I’m hoping [my decision] will be good for the moment.”

#### #2: Means of delaying acceptance of harsh realities

When parents are not ready to accept a harsh possibility or reality, the vagueness of faith, hope, and belief in miracles allows them to delay acceptance by introducing alternative possibilities. In our study, this took the form of parents who refused to withdraw care or participate in discussions about negative prognosis, citing beliefs that medical data and the physician’s recommendation were limited. First, R&S eliminate limits, because parents who believe in miracles need not accept the limits of medicine [58]. HCPs may say nothing more can be done for the child, but God is not limited by medical technology or natural laws (e.g. “God’s not like man. He can do things that we can’t do.”). Thus, R&S represent possibility for parents who need alternatives to a harsh reality. Second, R&S engender a sense of control over intolerable chaos [9]. Parents may believe that the child’s wellness is contingent on their continued expectation of a good outcome [59, 60]. One father felt his role in his child’s fight for survival was to stay positive and continue believing a miracle could occur. Therefore, maintaining hope and faith becomes a parental duty. In this way, faith-based hope is not only a justification to refute poor prognosis, but also an attempt to modify it.

When R&S function in this way, parents may tend to delay decisions or reject clinical recommendations, which can cause conflict [61]. In our study, medical information was usually not helpful in convincing these parents of poor prognosis. Parents who believe maintaining faith in divine healing is a spiritual duty may even see over-emphasis on medical evidence as a test of their faith (temptation to abandon faith in God), setting up a false dichotomy between faith and medicine [24]. Attempting to force a parent to make a decision they want to delay without addressing root of their avoidance may dismantle their coping system without putting anything in its place [62, 63].

#### #3: Foundation for Trust—In god, physicians, and self

While parents in this study often contrasted faith and medicine, they also often spoke of the ways God worked with and through people in the world. Much of the focus of divine intervention is on supernatural manifestations, like miracles, but most believers (including those in our study) would agree that the primary way God acts in the world is through natural means, including medicine [64]. The belief that God is behind the goings-on of the world provides an added sense of security to help parents cope with forces beyond their control. While Model #1 describes the sense of security parents gain from the belief that their decision need not be perfect, parents may alternatively be empowered by the belief that God authorizes and equips them to make the ‘right’ decision. The situations and decisions that parents and other caregivers face may feel so much larger than their capacity to make decisions, that they are paralyzed without the sense that God is guiding them.

In part, parents’ trust of HCPs and therapies was rooted in a trust of God; God brought the family to the hospital, God equipped the doctors with training and experience, God blessed the therapy, etc. When parents discussed the role of faith in decision-making, they emphasized their dual trust in the HCPs and in God (e.g. “[We] just put our faith in [the hospital] and God and see what happens.”). Parents surrender an incredible amount of control to health care teams when their children are seriously ill [56, 65]. Although studies differ on how important medical data is to parental decision-making, prior work shows that parents often consider the physician’s recommendation as one of (if not *the*) most important factors [3]. In our study, even the parents who fervently prayed for miracles reported that the physician’s recommendation was highly influential.

According to a meta-synthesis of feedback from parents facing end-of-life decisions, the degree to which parents trust physicians is partially based on their perceived hopefulness [3]. Similarly, our results suggest physicians without hope can appear to have “given up” on the child, leaving parents unable to trust their primary advisor. When parents’ trust of a physician is founded in R&S beliefs and values, lack of hope may send the message that God is not currently working through the physician [13].

Parents also need to trust their own judgment. Relative to HCPs who have clinical training and health care expertise, most parents are “unqualified” to make technical health care decisions. Although physicians are responsible for most care decisions, parents often have the final word in high-stakes decisions, including do-not-resuscitate orders. Parents may not feel they have authority to make life-and-death decisions, but knowledge God’s presence and divine plan (“I was meant to be [my child’s] mother”) makes them feel empowered.

### Impact on practice

Use of R&S in the ways we have described could play out in either positive or negative ways, and awareness of how R&S are functioning in a particular case could help HCPs improve communication, reduce conflict, and better support parental decision-making. We suggest the following practical observations and applications:

First, parental expression of R&S-motivated hope should not be construed as an expectation that the health care team do more than is technically possible. Religious hopes are often quite compatible with the acknowledgement of medical limitations (Model #1). When the limits of what can be offered with genuine benefit to a patient are reached, authoritative expression of this limit by HCPs does not preclude ongoing parental hope for a miracle [66].

Second, do not assume the vagueness of parental expressions of R&S belief is indicative of insincerity or total rejection of reality. The apparent vagueness of some belief systems makes room for possibilities that help families tolerate horrific situations while they adjust to new, unwanted realities (Model #2). When prognosis is dismal, appeal to God’s sovereignty may reflect that the seriousness of a situation is appreciated but unbearable without an external source of hope [56, 62]. Compassionate, competent care might dictate support of the family’s coping system, while employing strategies from within the family’s worldview to help them grow into the harsh reality [35].

Third, recognize when R&S are empowering parents to make hard decisions (Model #3), and do not seek to exclude “God” from discussions with these families. By affirming a family’s reliance on a higher power (rather than avoiding the topic), the HCP shows the parents that they can rely on God to guide both themselves and the health care team.

Finally, there are several strategies for helping parents participate in end-of-life decision-making while remaining faithful to their R&S belief systems. A chaplain familiar with a family’s religious tradition may offer stories from within the tradition that encourage acceptance of difficult realities, while preserving religious consolation (e.g. stories of faith in the face of unanswered prayer). HCPs can discuss the goals of parenting in light of faith that children are given by God, meaning parents are responsible for tending to them as gifts from God: both giving them good things and protecting them from harm. Avoiding an intervention that brings suffering without benefit is not likely to be construed as lack of faith, while failing to provide an intervention perceived as a potential good might be so construed.

### Limitations & future research

Our study’s primary limitation was that Christianity was the only faith tradition represented. Future research should examine the role of R&S when parents have a different R&S background or do not identify with a particular religion. Larger, more diverse studies may also allow for analysis of differences across race, ethnicity, and geographic setting, which would be especially valuable given the interaction of these factors with R&S. Additionally, the principal study targeted many decision-making factors, so matters pertaining to R&S were not fully explored in every interview. Research exclusively focusing on R&S could investigate several topics, including the effects of fervent belief in miracles on end-of-life decisions, how parents and HCPs communicate about R&S beliefs, and the role of hospital chaplains and other clergy in decision-making. Finally, our research demonstrates the need for the development of clinical and educational tools to help HCPs approach situations where R&S are important to families.

## Conclusion

The findings of this study show that the R&S concepts most central to parental decision-making are faith, hope, beliefs about God’s power and presence, prayer, and the possibility of miracles. The vague nature of these concepts and of parents’ descriptions of their impact on decision-making indicate that parents depend on the imprecision of R&S to make room for possibilities, and balance out limited medical realities. Thus, lack of clarity in parental expressions of R&S does not necessarily indicate insincerity or failure to appreciate the seriousness of the child’s medical prognosis. Recognizing the functions of R&S in a family’s decision-making process makes HCPs better equipped to support and communicate with parents, while providing the best possible care to their child.

## Additional file

Additional file 1: **Table S1.** Participants & interviews by case. **Table S2.** additional HCP quotes. (DOCX 24 kb)

## Abbreviations

CLTC: Complex life threatening conditions
HCP: Health care provider
HSCT: Hematopoietic stem cell transplant
NP: Nurse practitioner
R&S: Religion & spirituality (or religious & spiritual)
