# Supplementary material for: Exploring the vagueness of Religion & Spirituality in complex pediatric decision-making: a qualitative study
Source: BMC Palliat Care. 2018 Sep 12;17:107. doi: 10.1186/s12904-018-0360-y (PMC6134505; doi:10.1186/s12904-018-0360-y)
Supplement: Supplementary file 1 — Table S1. Participants & interviews by case. Table S2. additional HCP quotes. (DOCX 24 kb) [file 12904_2018_360_MOESM1_ESM.docx]

**Supplementary Tables**

**Table S1. Participants & Interviews by Case.**

| *Case* | *Parents  (# of interviews)* | *HCPs*  *(# of interviews)* | *Length of Case (infant’s status at completion)* |
| --- | --- | --- | --- |
| #1 | 1 Mother (1)  1 Father (3) | 3 Attending MDs (3), 1 Fellow MD (1), 1 Nurse (2), 1 Social Worker (3) | 213 days (deceased) |
| #2 | 1 Mother (10)  1 Father (11) | 1 Attending MD (2), 2 NP or PAs (2), 1 Nurse (1), 2 Social Workers (2) | 408 days (living) |
| #3 | 1 Mother (11)  1 Father (12) | 4 Attending MDs (4), 3 NP or PAs (6), 4 Nurses (5), 1 Social Worker (4) | 531 days (deceased) |
| #4 | 1 Mother (1)  1 Father (5) | 1 Attending MD (1), 1 NP or PA (2), 1 Nurse (3), 1 Social Worker (2) | 257 days (deceased) |
| #5 | 1 Mother (11)  1 Father (11) | 2 Attending MDs (3), 1 Fellow MDs (1), 2 NP or PAs (3), 1 Nurses (1), 2 Social Workers (2) | 388 days (living) |
| #6 | 1 Mother (12) | 1 Attending MDs (3), 3 NP or PAs (4), 1 Nurse (1), 1 Social Workers (3) | 380 days (living) |
| #7 | 1 Mother (9) | 1 Attending MDs (2), 1 NP or PA (2), 1 Nurse (1), 1 Social Workers (2) | 501 days (living) |
| #8 | 1 Mother (2)  1 Father (2) | 1 Attending MDs (1), 1 Fellow MDs (1), 1 NP or PAs (1), 2 Nurses (2), 1 Social Workers (2) | 8 days (deceased) |
| #9 | 1 Mother (8)  1 Father (8) | 1 Attending MDs (2), 2 NP or PAs (2), 1 Nurses (1), 1 Social Workers (2) | 387 days (living) |
| #10 | 1 Mother (5)  1 Father (5) | 1 Attending MDs (2), 1 NP or PAs (1), 1 Nurses (2), 2 Social Workers (4) | 341 days (deceased) |
| #11 | 1 Mother (3)  1 Father (3) | 2 Attending MDs (3), 1 Fellow MDs (1), 1 Nurses (4), 1 Social Workers (2) | Twin A: 19 days (deceased)  Twin B: 5 days (deceased) |
| #12 | 1 Mother (12)  1 Father (6) | 1 Attending MDs (2), 2 NP or PAs (2), 1 Nurses (1), 1 Social Workers (2) | 475 days (living) |
| #13 | 1 Mother (14)  1 Father (14) | 3 Attending MDs (4), 3 NP or PAs (4), 3 Nurses (4), 1 Social Workers (2) | 380 days (living) |
| #14 | 1 Mother (11) | 3 Attending MDs (3), 1 NP or PAs (1), 1 Nurses (1), 2 Social Workers (2) | 409 days (living) |
| #15 | 1 Mother (1) | 1 Attending MDs (1), 1 NP or PAs (1), 1 Nurses (1), 1 Social Workers (1) | 10 days (deceased) |
| #16 | 1 Mother (10)  1 Father (9) | 2 Attending MDs (5), 1 Fellow MDs (1), 2 NP or PAs (3), 3 Nurses (4), 3 Social Workers (4) | 432 days (living) |
|  |  |  |  |

**Table S2. Additional HCP Quotes.**

| HCP Category | Exemplary Quotes |
| --- | --- |
| Attending Physicians | “The family didn’t want to lose hope and I didn’t want to lose hope, until the final moment when, after an hour and a half resuscitation, his heart was coming back again and again. But his pulses were very poor, his perfusion was terrible, and it was very clear that at that time doing any more resuscitation is just hurting him in a sense of not *hurting* him ‘hurting him’—in terms of causing him pain—but it just prolonged the inevitable.”  *What do you think influenced these parents’ decision?* “Their faith. I think that’s what they kept telling us, … ‘People keep wanting me to terminate pregnancies and pull the plug on this child, but this is not for you to decide’ … and that she wouldn’t survive the pregnancy—‘Look, here she is! He said she couldn’t breathe off the ventilator, and now you’re telling me she maybe can.’ So I think that was undermining their faith in us, but I don’t think they had anywhere near as much faith in us anyway. But it was definitely faith for them.”  “I think at the end the parents felt like everything was done. They felt like, ‘Okay, this is God’s will to take the child,’ at that point.”  “I think there are certain belief systems, certain cultures, that might approach an identical situation dramatically different. I think that for the most part the biggest role that I’ve seen for spirituality and for that arena in health care is that it has generally been a comfort to families at times of great difficulty. And the people that are clergy or lay clergy, I think have had extended themselves remarkably to help families get through what are awful times sometimes.” |
| Fellow Physicians | “And then at times also they had been having prayers at the bedside, so it seemed to me that their faith was a big part of their decision-making as well.”  “That was made explicitly clear from the very beginning, that the patient—barring a miracle (which were the exact words stated)—would need a transplant to survive, but that we could give him medicines to keep him alive in the interim.”  “I’m a very spiritual person, so I believe being a physician is my calling and I’m interested in medical missions. I think it’s just a part of who I am, my innate nature is to incorporate that into how I interact with people. So I’m not preaching to anyone, but I think that does come across in my interactions and the decisions I make for my patients.” |
| Nurse Practitioners | “I know they have a very strong faith. I think they were putting [the child] into the hands of God. [That] is where I thought Mom was coming from. She was a very spiritual person and that’s what struck me—that they really had a lot of faith in [their child.]”  “I think once we get over that, then you’ll see her true potential of being able to come off the vent and ultimately get decannulated. I don’t know, there’s something about her. She’s destined for great things.”  “They maintained that they had faith and that [the child] was so tough that they would just leave it in God’s hands, pretty much.”  “I am quick to tell people that without a doubt I belief in God. Now, I don’t broach the subject with them unless they do, because I don’t know what people’s beliefs are. But if they bring it up, I am very quick to tell them, I too have a belief in God and that I believe that miracles do happen. But we need to understand that [this is] reality.”  “I do have faith, but I don’t bring it up first. I think it plays a big role in [decision-making]. But not to the point where I would have a problem with withdrawing support on a child at all. I completely believe in palliative care and withdrawal of support.” |
| Nurses | “It’s kind of like an oxymoron when someone says to you, ‘Well, we’ll let God take care of it.’ Hey, if God took care of it, your baby wouldn’t be here.”  “Their response is, God has this—even when the echo and [the doctor] was saying, ‘We don’t have any cases of anyone surviving, but you never know.’ And Mom’s response was like, ‘You’re right, because it’s not in our hands. None of this is in our hands.”  “I know she’s a strong Christian and believes in prayer and you know I think she just placed it in the Lord’s hands.”  “A lot of the time, a parent’s going to think, ‘It’s wrong for me to want my child to die. I can’t tell you to let my child die.’ But, we need to get them on the thought of it’s also wrong to let them suffer” |
| Social Workers | “So that’s how they viewed this, is that she’s a beautiful miracle from God and God has a, you know, a path for her and we have no idea what that will be. I think they know that she’s sick and they understand that she needs this medicine to keep her heart open, but I think they also think that God could work a miracle, and heal her or cure her.”  “I think the religious part of it can bring in some guilt, too. And we didn’t go there with them very much, as far as Catholicism. They both pray and have belief, so that’s another piece of it.”  “I would [use] the family’s God talk, as you have to cater to them—‘allow God to decide when her time is….and remove the things that are keeping her, and allow God to then see, because we might be able to stop the prostaglandin, and she may still be here a day, a week, a month, but God will decide when that is to happen.’”  “There are times when the dialogue shuts down because a family is labeled as very primitively religious. They may believe in miracles. Well, that doesn’t mean that they might not use miracles as metaphors [or] see a different thing as the miracle. And it scares me when we decide that it’s going to be impossible to actually have a dialogue. I feel it’s my role to make sure family and staff know that the dialogue has to be ongoing in these kinds of situations and not just that because it’s been said once that it is the end of the discussion.” |
|  |  |
